# Complete Genome Sequences of Two *Rhodobacter* Strains

**DOI:** 10.1128/MRA.01162-18

**Published:** 2018-09-27

**Authors:** Brian P. Anton, Richard J. Roberts, Alexey Fomenkov, Allison Humbert, Natalie Stoian, Jill Zeilstra-Ryalls

**Affiliations:** a New England Biolabs, Ipswich, Massachusetts, USA; b Bowling Green State University, Bowling Green, Ohio, USA; Indiana University Bloomington

## Abstract

We report the complete genome sequences of two strains of the *Alphaproteobacteria* genus Rhodobacter, Rhodobacter blasticus 28/5, the source of the commercially available enzyme RsaI, and a new isolate of Rhodobacter sphaeroides 2.4.1. Both strains contain multiple restriction-modification systems, and their DNA methylation motifs are included in this report.

## ANNOUNCEMENT

Several type II restriction endonucleases (REases) have been isolated from strains of Rhodobacter (formerly known as *Rhodopseudomonas*) sphaeroides (class *Alphaproteobacteria*). RsaI was first purified from strain 28/5 (1); RshI, an isoschizomer of PvuI, was first purified from the type strain 2.4.1 (ATCC 17023) (2); and RsrI, an isoschizomer of EcoRI (3, 4), and RsrII were both purified from the wild-type strain RS630 (5).

We have sequenced and characterized the methylomes of Rhodobacter strains 28/5 and 2.4.1, both originally obtained from the laboratory of Samuel Kaplan (University of Texas Health Science Center––Houston), using the PacBio RS II platform. Both strains were cultured aerobically to an optical density at 660 nm (OD_660_) of 0.8, with strain 2.4.1 cultured in Sistrom’s minimal medium A with succinate (6) and strain 28/5 cultured in LB (7). Total DNA was isolated using the GenElute bacterial genomic DNA kit (Sigma-Aldrich) and fragmented to roughly 10 to 20 kb with g-TUBES (Covaris). Standard 20-kb libraries were prepared and sequenced with P6-C4 chemistry using two single-molecule real-time (SMRT) cells each, for one of which the library was size selected (9 to 50 kb) using the BluePippin electrophoresis system (Sage), and movie times of 240 or 360 min. Sequencing reads (182,877, mean subread length 6,892 for 2.4.1; 79,991, mean subread length 4,023 for 28/5) were processed, mapped, and assembled *de novo* with RS_HGAP_Assembly.3 (8) in the SMRT Analysis 2.3.0 environment using default settings. Error correction and closure were performed using RS_BridgeMapper.1, and methylation patterns were determined using RS_Modification_and_Motif_Analysis.1, both also within SMRT Analysis. Annotation was performed at the NCBI using PGAP (9).

Strain 28/5 assembled as a single chromosome of 3.54 Mb (66.6% G+C content) and a single plasmid of 157 kb (62.4% G+C content). Consistent with a previous report suggesting that it was not in fact an R. sphaeroides strain (10), we found the 16S rRNA gene sequences of 28/5 to be 99% identical (1,377/1,389) to those of Rhodobacter blasticus DSM 2131 (synonym, ATCC 33485), a type strain. We suggest that the RsaI source strain be renamed R. blasticus strain 28/5.

R. sphaeroides 2.4.1 assembled as two chromosomes of 3.19 Mb and 943 kb (both with 69.0% G+C content) and four plasmids, A (124 kb), B (114 kb), C (107 kb), and D-E (152 kb). There is substantial agreement between our sequence and the Department of Energy (DOE) reference (GenBank accession numbers CP000143 through CP000147, DQ232586, and DQ232587), although our assembled plasmid D-E is a combination of the DOE reference plasmid E and fragment D (DQ232587), and our plasmid A is a slightly larger, circularized version of the DOE fragment A (DQ232586). Most indels were 1 to 2 bp long and in mononucleotide runs, and most single nucleotide polymorphisms (SNPs) were concentrated in a small number of genes that are imperfectly duplicated within the genome. The long reads inherent in the PacBio platform often enable the correct assembly of such duplicated regions.

Table 1 shows the methylated motifs identified in these two strains and the responsible methyltransferases (MTases). Methylation data have been deposited in GenBank and REBASE (11).

**TABLE 1:** Methylated motifs in *R. blasticus* strain 28/5 and *R. sphaeroides* strain 2.4.1

Strain	Motif^*a*^	Modification	% motifs detected	Predicted MTase locus
28/5	GANTC	m6A	98.35	09535 (M.RsaII)
28/5	RCGCCTG	m4C	91.11	15910
28/5	GTAC	m4C	87.65	04320 (M.RsaI)
28/5	RGATCY	m4C	36.4^*b*^	06345 (M.RsaIII)
2.4.1	GANTC	m6A	99.51	00245 (M.Rsp241II)
2.4.1	CGATCG	m5C	∼5^*c*^	19975 (M.Rsp241I)

a Locations of methylated bases on the top strand (A or C) and bottom strand (T or G) are underlined.

b Manifested as several related sites using the automated motif-calling pipeline. This motif was deduced from manual analysis.

c Manifested as CGATCGVR, modified at 37.47%. The gene responsible is an m5C MTase, and its exact recognition profile has been determined separately (B. P. Anton, unpublished data).

### Data availability.

The DDBJ/ENA/GenBank accession numbers for R. blasticus 28/5 are CP020470 and CP020471 and for R. sphaeroides 2.4.1 are CP030271, CP030272, CP030273, CP030274, CP030275, and CP030276. The Sequence Read Archive (SRA) accession numbers for R. blasticus 28/5 and R. sphaeroides 2.4.1 are SRP158375 and SRP157902, respectively.
